# Gene Expression Profiles of Human Dendritic Cells Interacting with *Aspergillus fumigatus* in a Bilayer Model of the Alveolar Epithelium/Endothelium Interface

**DOI:** 10.1371/journal.pone.0098279

**Published:** 2014-05-28

**Authors:** Charles Oliver Morton, Mirjam Fliesser, Marcus Dittrich, Tobias Mueller, Ruth Bauer, Susanne Kneitz, William Hope, Thomas Richard Rogers, Hermann Einsele, Juergen Loeffler

**Affiliations:** 1 University of Western Sydney, School of Science and Health, Sydney, Australia; 2 Universität Wuerzburg, Medizinische Klinik & Poliklinik II, WÜ4i, Wuerzburg, Germany; 3 Department of Bioinformatics, Biocenter, University of Wuerzburg, Wuerzburg, Germany; 4 Department of Molecular and Clinical Pharmacology, University of Liverpool, Liverpool, United Kingdom; 5 Department of Clinical Microbiology, Sir Patrick Research Laboratory, Trinity College Dublin, Dublin, Ireland; David Geffen School of Medicine at University of California Los Angeles, United States of America

## Abstract

The initial stages of the interaction between the host and *Aspergillus fumigatus* at the alveolar surface of the human lung are critical in the establishment of aspergillosis. Using an *in vitro* bilayer model of the alveolus, including both the epithelium (human lung adenocarcinoma epithelial cell line, A549) and endothelium (human pulmonary artery epithelial cells, HPAEC) on transwell membranes, it was possible to closely replicate the *in vivo* conditions. Two distinct sub-groups of dendritic cells (DC), monocyte-derived DC (moDC) and myeloid DC (mDC), were included in the model to examine immune responses to fungal infection at the alveolar surface. RNA in high quantity and quality was extracted from the cell layers on the transwell membrane to allow gene expression analysis using tailored custom-made microarrays, containing probes for 117 immune-relevant genes. This microarray data indicated minimal induction of immune gene expression in A549 alveolar epithelial cells in response to germ tubes of *A. fumigatus*. In contrast, the addition of DC to the system greatly increased the number of differentially expressed immune genes. moDC exhibited increased expression of genes including *CLEC7A, CD209* and *CCL18* in the absence of *A. fumigatus* compared to mDC. In the presence of *A. fumigatus*, both DC subgroups exhibited up-regulation of genes identified in previous studies as being associated with the exposure of DC to *A. fumigatus* and exhibiting chemotactic properties for neutrophils, including *CXCL2, CXCL5, CCL20*, and *IL1B*. This model closely approximated the human alveolus allowing for an analysis of the host pathogen interface that complements existing animal models of IA.

## Introduction

*Aspergillus fumigatus* is a ubiquitous mould that is generally found in soil or decaying vegetation [1]. It produces vast numbers of spores (conidia) which readily become airborne to aid dispersal and their small size, app. 2.5–3.5 µm in diameter, allows them to enter the lung alveoli [2]. Depending on the immune status of the host, *Aspergillus* species are responsible for a spectrum of diseases in humans [3]. In patients with haematological malignancies who undergo allogeneic stem cell transplantation, invasive aspergillosis (IA) is the leading infective cause of death [4].

The human lung alveolar surface is the initial site of interaction between the host and the fungus. Inhaled resting conidia that reach the alveoli are inert to the immune system [5]. Ordinarily germinating conidia encounter a number of pulmonary defenses including respiratory mucus, antimicrobial molecules, such as defensins, and innate immune cells such as pulmonary macrophages and dendritic cells [6]. Furthermore, the alveoli, which consist of an epithelial cell layer and extracellular matrix surrounded by capillaries, may play an important role in host innate immunity [7], [8]. Understanding these initial events of IA is of great importance to fully characterize the pathogenesis and to identify potential novel therapeutic strategies. Increasingly complex tissue culture models have assisted in the understanding of fungal infections at epithelial barriers, especially for *Candida* [9]. It was shown that proteases were important for inducing a pro-inflammatory response in epithelia [10] and that polymorphonuclear cells added to the model reduced tissue damage and more closely mimicked the *in vivo* situation [11]. A model of the human alveolus consisting of a bilayer of human epithelial and endothelial cells was used to monitor the invasion of alveolar epithelium by *A. fumigatus* [6], [12].

In epithelial models and direct interactions between *A. fumigatus* and monocytes [13], [14], dendritic cells [15], 16 and neutrophils [17] gene expression analyses have indicated the importance of a host Th1 pro-inflammatory response. The importance of a pro-inflammatory response in defense against *A. fumigatus* suggests a direct role for dendritic cells (DC), which act as sentries of the innate immune system [18]. A network of DC is present in the lungs and at mucosal surfaces of most tissues where they sample their immediate microenvironment to detect pathogenic microbes [19]. Pulmonary DC phagocytose microbes and through cytokine signalling mature during migration to the lymph nodes where they present microbial antigens to activate T-cell populations [20]. In addition to antigen presentation it was found that pulmonary retention of inflammatory DC in a murine model of IA was correlated to improved outcomes [21]. Without immunomodulatory signaling from neutrophils there was increased recruitment of DC to the lungs in the neutropenic mice, these DC were probably able to assist in compensating for the absence of neutrophils [21]. It has been observed that DC can distinguish between hyphae and conidia leading to rapid killing of hyphae but extended survival of conidia within the phagosome [15], [20]. There are different DC subsets present in tissues and may induce different kinds of immune responses and determine the outcome of infection. In addition, for *Bacillus anthracis* spores, it has been shown that primary antimicrobial defenses are orchestrated by lung epithelial cells interacting in very close association with CD11c+ DCs, which phagocytose spores and transport them to lymph nodes [22], [23].

In this study, a previously described bilayer model of the human alveolus [8], which was developed for pharmacological studies, was adapted to analyze expression profiles of immune-relevant genes in different subsets of DC interacting with *A. fumigatus*. To date, immune cell and *A. fumigatus* interaction experiments have been performed in planktonic culture. This model was chosen to better approximate how immune cells would interact with *A. fumigatus* in a local lung environment, at the alveolar interface where early infection occurs. Experiments included two relevant DC subpopulations, myeloid DC (mDC) and monocyte-derived DC (moDC). mDC, which carry CD1c as a characteristic marker were isolated from peripheral blood *ex vivo* and perform complex functions, including production of various chemokines. The *in vitro* generated moDC were produced under standardized conditions and in large quantities; these cells are regularly used in immunotherapy protocols. The major goals of this research were to analyze if this bilayer model was suitable for expression profiling studies to approximate local early IA *in vitro* and if moDC and mDC subpopulations interacting with *A. fumigatus* in this setting differ in their gene expression profiles.

## Results

### The human alveolar bilayer model is suitable for gene expression profiling experiments

The average yield of total RNA from the epithelial layer was 71.5 (±16) ng µl^−1^ and from the endothelial layer was 41.2 (±17) ng µl^−1^. The RIN (RNA integrity) values were constantly >8 (data not shown). Consequently, these numbers indicate that the RNA extracted from the bilayer model system was of sufficient quantity and quality, which is a major prerequisite for microarray analyses.

**Figure 1:**
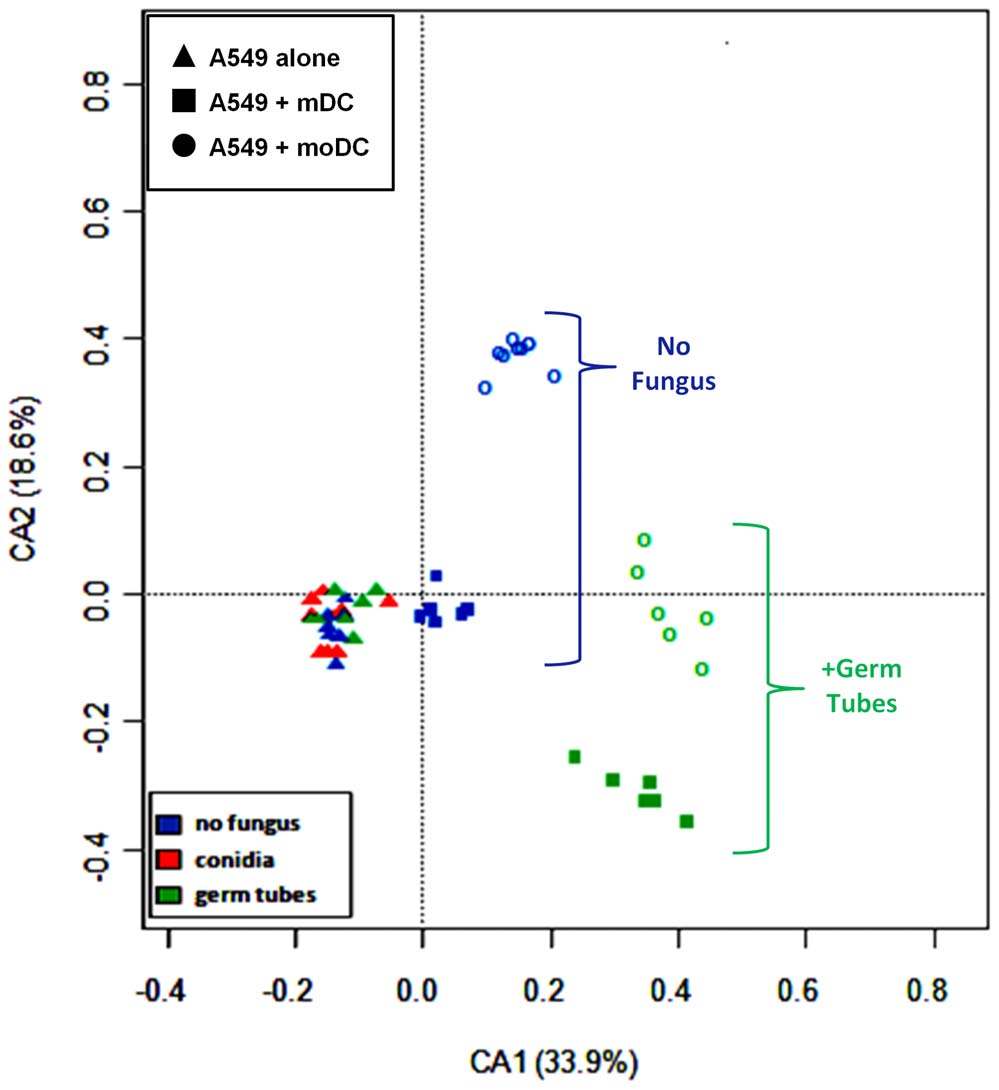
Correspondence analysis indicating the factors with the greatest influence on the microarray data. The triangles represent the A549 cells and these clustered closely even in the presence of *A. fu*migatus germ tubes. Whereas mDC, represented by squares, clustered close to the A549 data except when germ tubes were added which caused a distinct cluster to be formed. The moDC, represented by circles, formed two distinct clusters one consisting of datasets without fungus. The second cluster for datasets with germ tubes was closer to the mDC dataset with germ tubes. These data indicate that including moDC with the A549 layer leads to greater changes in gene expression compared to the A549 layer than in the absence of exogenous immunostimulators such as *A. fumigatus*. The data indicate that conidia are not strong immunostimulators of A549 cells. The addition of germ tubes caused similar gene expression responses when mDC or moDC were present.

Gene expression profiles were based on a tailored array, which was designed according to previous results from a genome-wide expression array analyses in DCs [16]. In a basic initial analysis, genes that had log fold change values (ratios of target gene to house-keeping gene expressed as log_2_ values) of <−0.5 or >1.0 and a p-value <0.05 were considered significantly differentially expressed for analysis. Correspondence analysis of the array data indicated that the most important factors influencing differential gene expression are the fungus and moDC (Figure 1).

**Figure 2:**
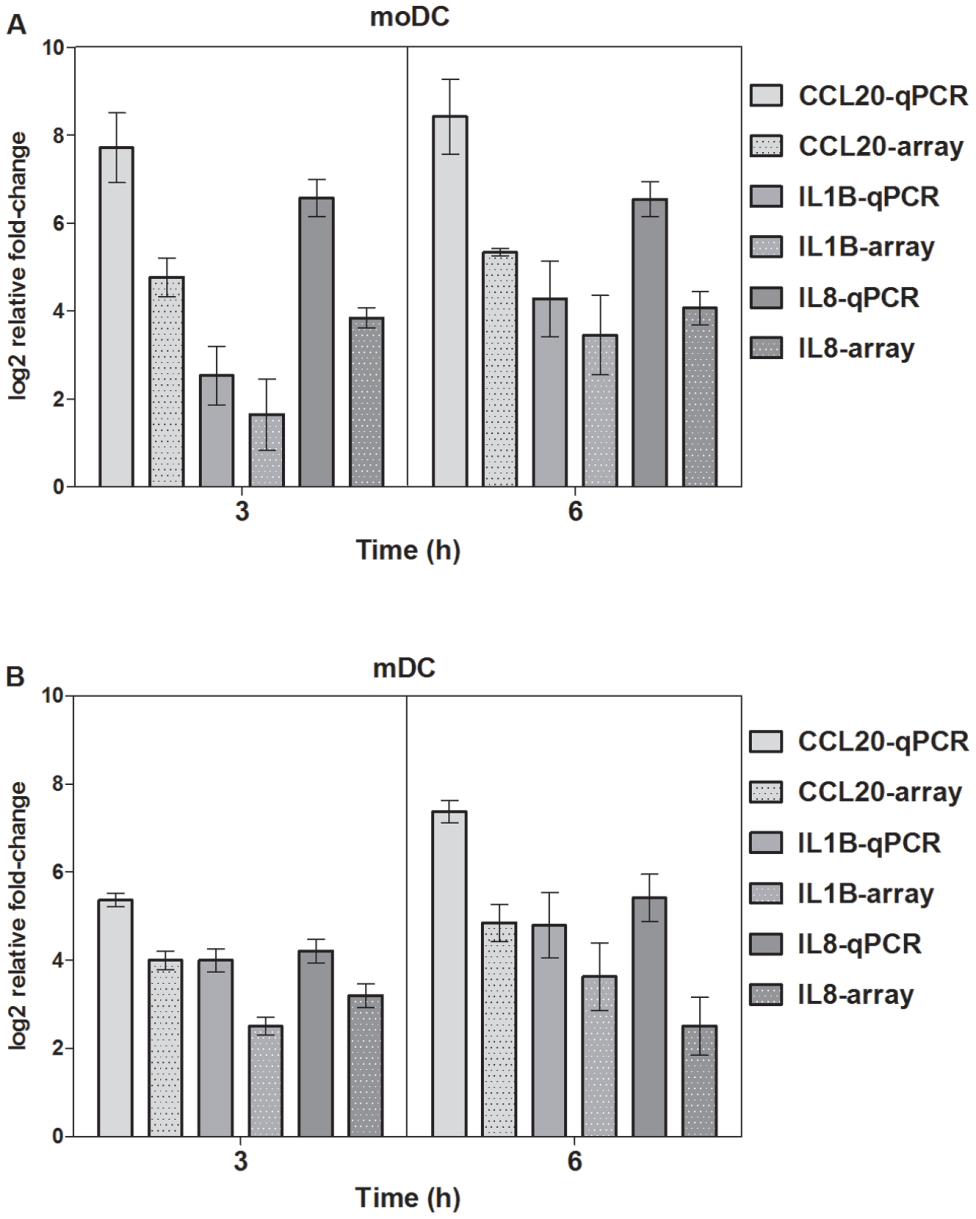
RT-qPCR validation of differential gene expression observed in microarray experiments measuring cellular interactions involving (a) moDC and (b) mDC. Gene expression data from the immune gene microarray shown as mean log_2_ values of ratios of relative expression determined by RT-qPCR compared to array data. Error bars in both charts indicate the standard error.

To confirm the relative fold change values obtained by microarray analyses, we performed RT-qPCR assays for genes showing the greatest differential expression on the array. All genes tested showed similar expression patterns for both RT-qPCR and microarray assays (Figure 2). Thus, RT-qPCR results supported the data generated by the microarray analysis.

### Gene expression analysis in structural cell layers of the alveolar bilayer model

No genes were differentially regulated in the HPAEC cell layer after 3 h and 6 h of exposure to conidia. The response of the A549 cell layer was more dynamic (Table 1) but of 15 differentially regulated genes only *GSK3A* showed up-regulation. Similarly the addition of germ tubes caused the differential regulation of 10 genes in the A549 cell layer with only *SOD1* showing up-regulation (Table 2). These data indicated that positive regulation of immune genes in this model suggests only limited interaction between the fungus and the epithelial cell layer A549.

**Table 1:** Gene Expression in A549 epithelial cell layer induced by addition of *A. fumigatus* conidia.

		Fold Change	
Gene	Gene Name	3 h	6 h
CCL2	Chemokine (C-C-Motif) Ligand 2	−1.28	−1.47
CCL17	Chemokine (C-C-Motif) Ligand 17	−1.43	-
CCL19	Chemokine (C-C-Motif) Ligand 19	−1.55	−1.29
CCL21	Chemokine (C-C-Motif) Ligand 21	-	−1.43
CCL23	Chemokine (C-C-Motif) Ligand 23	-	−1.69
CCL25	Chemokine (C-C-Motif) Ligand 25	-	−1.27
CXCL3	Chemokine (C-X-C-Motif) Ligand 3	−1.40	−1.07
CXCL6	Chemokine (C-X-C-Motif) Ligand 6	−1.56	−1.54
CXCL9	Chemokine (C-X-C-Motif) Ligand 9	−1.27	−1.39
DCSIGN	Dendritic Cell-Specific Intercellular adhesion molecule-3-Grabbing Non-integrin	-	−1.45
Dectin1	Dectin-1	-	−1.31
GSK3a	Glycogen synthase kinase 3 alpha	**1.42**	-
LTA	Lymphotoxin alpha	-1.82	-
NFKBIA	nuclear factor of kappa light polypeptide gene enhancer in B-cells inhibitor, alpha	-	−1.11
PECR	peroxisomal trans-2-enoyl-CoA reductase	-	−1.37

Expression is expressed as Fold Change (Log_2_); ratio of gene expression in cells plus *A. fumigatus* relative to cells minus *A. fumigatus*.

**Table 2:** Gene Expression in A549 epithelial cell layer induced by addition of *A. fumigatus* germ tubes.

		Fold Change	
Gene	Gene Function	3 h	6 h
MPO	Myeloperoxidase	−0.75	−0.91
NCF-1	Neutrophil cytosolic factor 1	−0.97	−0.93
NFKBIL1	nuclear factor of kappa light polypeptide gene enhancer in B-cells inhibitor-like 1	−1.08	−1.03
NFKB2	Nuclear factor of kappa light polypeptide gene enhancer in B-cells 2	−0.57	−0.89
PTX3	Pentraxin 3	−0.76	−0.81
SOD1	Superoxide dismutase 1	−	**1.75**
TLR1	Toll-like Receptor 1	−0.88	−0.80
TLR3	Toll-like Receptor 3	−0.59	−0.68
TLR5	Toll-like Receptor 5	−0.52	−0.69
TLR7	Toll-like Receptor 7	−0.68	−0.61

Expression is expressed as Fold change (Log_2_); ratio of gene expression in cells plus *A. fumigatus* relative to cells minus *A. fumigatus*.

### Gene expression analysis of DC interacting with A. fumigatus

The expression of immune genes from DCs interacting with *A. fumigatus* were measured as a control to compare to the results obtained from the interaction experiments on the bilayer model (Table 2 and Table 3). Exposure to conidia caused the up-regulation of seven genes (total of 13 differentially regulated) in mDC and eight genes (total of eight differentially regulated) in moDC (Table 3). This was greater than the number of up-regulated genes observed in the A549 cells exposed to the same stimulus (Table 1). Exposure to germ tubes caused a greater degree of differential regulation than conidia with up-regulation of nine genes (from a total of 19) in mDC and 10 genes (from a total of 17) in moDC (Table 4). This reflects the greater immunogenicity of germ tubes and indicates a similar response by both subsets of DC to *A. fumigatus*.

**Table 3:** Gene expression of immune-related genes in donor myeloid dendritic cells or monocyte-derived dendritic cells with the addition of *A. fumigatus* conidia (after 6 hours incubation).

		Fold Change	
Gene	Gene Name	mDC	moDC
CCL2	Chemokine (C-C-Motif) Ligand2	**2.68**	
CCL4	Chemokine (C-C-Motif) Ligand 4	**2.24**	**3.46**
CCL5	Chemokine (C-C-Motif) Ligand 5	**1.72**	
CCL7	Chemokine (C-C-Motif) Ligand 7	**2.22**	**1.5**
CCL20	Chemokine (C-C-Motif) Ligand 20		**1.7**
CCR2	Chemokine (C-C-Motif) Receptor 2	−1.23	
CXCL1	Chemokine (C-X-C-Motif) Ligand 1	**1.97**	
CXCL2	Chemokine (C-X-C-Motif) Ligand 2		**4.1**
CXCL5	Chemokine (C-X-C-Motif) Ligand 5	**2.29**	
IL1A	Interleukin-1 alpha	**1.82**	**1.2**
IL1B	Interleukin-1 beta		**3.35**
IL8	Interleukin-8		**1.8**
IL8RB	Interleukin-8 Receptor B	−0.92	
IL10RA	Interleukin-10 Receptor A	−1.44	
IL10RB	Interleukin-10 Receptor B	−1.00	
NCF-1	Neutrophil Cytosolic Factor 1	−0.93	
NFKBIA	Nuclear factor of kappa light polypeptide gene enhancer in B-cells inhibitor, alpha		**2.49**
TNFRSF1A	Tumour necrosis factor-receptor superfamily 1A	−1.51	−0.74

Expression is expressed as Fold Change (Log_2_); ratio of gene expression in DC minus *A. fumigatus* relative to DC plus *A. fumigatus*.

**Table 4:** Gene expression of immune-related genes in donor myeloid dendritic cells or monocyte-derived dendritic cells induced by the addition of *A. fumigatus* germ tubes (after 6 hours incubation).

		Fold Change	
Gene	Gene Function	mDC	moDC
CCL1	Chemokine (C-C-Motif) Ligand 1	**1.53**	
CCL4	Chemokine (C-C-Motif) Ligand 4	**2.4**	**3.52**
CCL5	Chemokine (C-C-Motif) Ligand 5		**2.1**
CCL7	Chemokine (C-C-Motif) Ligand 7	**1.5**	
CCL20	Chemokine (C-C-Motif) Ligand 20		**2.51**
CCR5	Chemokine (C-C-Motif) Receptor 5	−1.69	
CD81	CD81-Molecule		−0.99
CXCL1	Chemokine (C-X-C-Motif) Ligand 1	**1.98**	
CXCL2	Chemokine (C-X-C-Motif) Ligand 2		**4.2**
CXCL3	Chemokine (C-X-C-Motif) Ligand 3	**2.2**	**1.11**
CXCL5	Chemokine (C-X-C-Motif) Ligand 5	**3.13**	
CXCL6	Chemokine (C-X-C-Motif) Ligand 6	**1.05**	
CXCL13	Chemokine (C-X-C-Motif) Ligand 13	−1.13	
DCSIGN	Dendritic Cell-Specific Intercellular adhesion molecule-3-Grabbing Non-integrin		−2.1
GSK3a	Glycogen synthase kinase 3 alpha		−0.53
IL1A	Interleukin-1 alpha	**1.92**	**1.53**
IL1B	Interleukin-1 beta		**3.3**
IL8	Interleukin-8	**2.1**	**2.2**
IL1R1	Interleukin-1 Receptor 1		−0.58
IL10RA	Interleukin-10 Receptor A	−1.43	
IL10RB	Interleukin-10 Receptor B	−1.33	−0.72
MYD88	Myeloid differentiation primary response gene (88)	−0.68	
NCF-1	Neutrophil Cytosolic Factor 1	−0.93	
NFKBIA	Nuclear factor of kappa light polypeptide gene enhancer in B-cells inhibitor, alpha		2.4
PSMA7	Proteasome (prosome, macropain) subunit, alpha type, 7	−0.59	
TNF	Tumour necrosis factor	**1.56**	**2.3**
TNFRSF1A	Tumour necrosis factor-Receptor Superfamily 1A	−1.52	−1.0
TXN	Thioredoxin		**1.32**

Expression is expressed as Fold Change (Log_2_); ratio of gene expression in DC plus *A. fumigatus* relative to DC minus *A. fumigatus*.

### Gene expression analysis with the addition of DCs

To mimic the *in vivo* situation more specified, mDC or moDC were added to the bilayer model. The rationales to compare these two DC types were that (i) we already published data on gene expression analysis with moDC co-cultured with *A. fumigatus* and therefore are able to compare our results to the bilayer model system and (ii) different DC sub-populations may induce different immune responses, e.g., Th1 or Th17 types.

The addition of either mDC or moDC to the bilayer model greatly increased the number of up-regulated genes (Table 5) compared to the gene expression observed in the A549 layer plus *A. fumigatus* (Tables 1 and 2). The addition of mDC led to the differential regulation of 12 genes (five up-regulated) whereas the addition of moDC caused differential regulation of 27 genes (nine up-regulated) (Table 5). Interestingly mDC showed greater expression of *IL1B* compared to moDC, which expressed more *CLEC7A*, *CD209* and 30% more C-C-motif chemokine ligands. This indicated that the moDC were in a state of greater regulatory excitation than mDC, which may be expected since moDC were induced to become moDC through exposure to cytokines *in vitro* whereas the mDC were primary immune cells isolated directly from the peripheral blood.

**Table 5:** Gene Expression in A549 epithelial cell layer and donor myeloid dendritic cells or monocyte-derived dendritic cells without the addition of *A. fumigatus* germ tubes (after 6 hours incubation).

		Fold Change	
Gene	Gene Name	mDC	moDC
CCL4	Chemokine (C-C-Motif) Ligand 4		**1.114**
CCL13	Chemokine (C-C-Motif) Ligand 13		**4.904**
CCL17	Chemokine (C-C-Motif) Ligand 17	**3.816**	**3.067**
CCL18	Chemokine (C-C-Motif) Ligand 18		**5.349**
CCL23	Chemokine (C-C-Motif) Ligand 23		**1.669**
CCL24	Chemokine (C-C-Motif) Ligand 24	**1.616**	
CXCL5	Chemokine (C-X-C-Motif) Ligand 5	**1.201**	
CXCL10	Chemokine (C-X-C-Motif) Ligand 10	−0.576	
CXCR4	Chemokine (C-X-C-Motif) Receptor 4	**1.894**	**1.520**
Dectin1	Dectin - 1		**1.557**
DCSIGN	Dendritic Cell-Specific Intercellular adhesion molecule-3-Grabbing Non-integrin		**2.402**
GSK3a	Glycogen synthase kinase 3 alpha		−1.156
HSPA8	Heat shock 70 kDa protein 8		−1.271
IL1A	Interleukin-1 alpha	−0.627	
IL1B	Interleukin-1 beta	**1.250**	
IL1R1	Interleukin-1 Receptor 1	−0.710	−0.736
IL6R	Interleukin-1 Receptor 6	−0.511	
IL8RA	Interleukin-8 Receptor A		−0.631
IL11RA	Interleukin-11 Receptor A		−0.834
IL12B	Interleukin-12 Receptor B		−0.723
IL23	Interleukin-23		−0.663
MYO1C	Myosin 1C	−0.766	−0.689
PECR	peroxisomal trans-2-enoyl-CoA reductase	−1.006	−1.022
PSMA7	proteasome (prosome, macropain) subunit, alpha type, 7	−0.904	−1.185
SOD1	Superoxide dismutase 1		−0.887
TLR1	Toll-like Receptor 1		−1.172
TLR2	Toll-like Receptor 2		−0.751
TLR3	Toll-like Receptor 3		−0.900
TLR5	Toll-like Receptor 5		−1.103
TLR7	Toll-like Receptor 7		−1.229
TNFRSF1A	Tumour necrosis factor-Receptor Superfamily 1A		−0.841
TNFRSF1B	Tumour necrosis factor-Receptor Superfamily 1B		**1.054**
TOLLIP	Toll interacting protein		−0.508
			

Expression is expressed as Fold Change (Log_2_); ratio of gene expression in cells plus DC relative to cells minus *DC*.

The introduction of *A. fumigatus* germ tubes onto the A549 cell layer along with DC induced a greater increase in the number of differentially expressed genes in treatments with mDC than in those with moDC (Table 6) compared to the treatments without germ tubes (Table 5). The number of differentially regulated genes was 42 (14 up-regulated) in mDC compared to 49 genes (17 up-regulated) in moDC. The significant up-regulation of *IL8* and *CCL20* in both types of DC was consistent with previous studies on the interaction between moDC and *A. fumigatus* [15], [16]. *IL1B* showed increased expression in mDC and moDC indicating the initiation of a pro-inflammatory response, the mDC value was more than two-fold greater than the response in moDC.

**Table 6:** Expression of immune-related genes in A549 epithelial cell layer and donor myeloid dendritic cells or monocyte-derived dendritic cells induced by the addition of *A. fumigatus* germ tubes (after 6 hours incubation).

		Fold Change	
Gene	Gene Function	mDC	moDC
ACTB	Actin B	-	**1.018**
ATP6V1A	ATPase, H+ transporting, lysosomal 70 kDa, V1 subunit A	−1.875	−1.616
CCL4	Chemokine (C-C-Motif) Ligand 4	**3.274**	**5.244**
CCL5	Chemokine (C-C-Motif) Ligand 5	**1.144**	**1.024**
CCL13	Chemokine (C-C-Motif) Ligand 13	-	**2.632**
CCL17	Chemokine (C-C-Motif) Ligand 17	-	**1.043**
CCL18	Chemokine (C-C-Motif) Ligand 18	-	**2.377**
CCL19	Chemokine (C-C-Motif) Ligand 19	−0.911	−0.718
CCL20	Chemokine (C-C-Motif) Ligand 20	**4.189**	**3.084**
CCL21	Chemokine (C-C-Motif) Ligand 21	−0.787	−0.772
CCL23	Chemokine (C-C-Motif) Ligand 23	**1.128**	-
CCL24	Chemokine (C-C-Motif) Ligand 24	-	−0.703
CCL26	Chemokine (C-C-Motif) Ligand 26	−0.873	−0.679
CCR4	Chemokine (C-C-Motif) Receptor 4	−0.894	−0.646
CCR5	Chemokine (C-C-Motif) Receptor 5	−0.557	−0.567
CD81	CD81-Molecule	−1.139	−0.918
CSF2	Colony stimulating factor 2 (granulocyte-macrophage)	-	**1.311**
CXCL1	Chemokine (C-X-C-Motif) Ligand 1	**3.071**	-
CXCL2	Chemokine (C-X-C-Motif) Ligand 2	**4.033**	**3.307**
CXCL3	Chemokine (C-X-C-Motif) Ligand 3	**2.216**	**1.009**
CXCL5	Chemokine (C-X-C-Motif) Ligand 5	**3.282**	**2.116**
CXCR4	Chemokine (C-X-C-Motif) Receptor 4	**1.111**	**1.799**
GOLGA4	Golgi autoantigen, golgin Subfamily a, 4	−0.504	-
GSK3a	Glycogen synthase kinase 3 alpha	−1.831	−2.737
HSPA8	Heat shock 70 kDa protein 8	-	−0.809
HSP90AB1	Heat shock protein 90 kDa alpha (cytosolic), class B member 1	-	−1.957
ICAM1	Intercellular adhesion molecule 1	**1.658**	**1.931**
IL1B	Interleukin-1 beta	**3.857**	**1.363**
IL1R1	Interleukin-1 Receptor 1	−0.514	−0.595
IL8	Interleukin-8	**1.894**	**2.268**
IL10RB	Interleukin-10 Receptor B	-	−0.638
IL11	Interleukin-11	-1.046	−0.968
IL11RA	Interleukin-11 Receptor A	−1.773	−1.724
IL12B	Interleukin-12 Receptor B	-	−0.512
MIF	Macrophage migration inhibitory factor	−0.880	−1.461
MYD88	Myeloid differentiation primary response gene (88)	−0.782	−1.337
MYO1C	Myosin 1C	−1.598	−1.471
NFKBIA	Nuclear factor of kappa light polypeptide gene enhancer in B-cells inhibitor, alpha	**1.262**	**1.377**
NIBP	Trafficking protein particle complex 9	−0.507	−0.564
PECR	Peroxisomal trans-2-enoyl-CoA reductase	−1.268	−1.048
PSMA7	Proteasome (prosome, macropain) subunit, alpha type, 7	−2.081	−2.079
PTX3	Pentraxin 3	**1.188**	**1.306**
SOD1	Superoxiddismutase 1	−2.049	−2.518
TLR2	Toll-like Receptor 2	−0.965	−1.521
TNFRSF1A	Tumour necrosis factor-Receptor Superfamily 1A	−2.886	−2.623
TOLLIP	Toll interacting protein	−1.357	−1.395
TXN	Thioredoxin	−2.049	**-**
USP49	Ubiquitin specific peptidase 49	−0.820	−0.695
ZNF710	Zinc-fingerprotein 710	−0.697	−0.807

Expression is expressed as Fold Change (Log_2_); ratio of gene expression in cells plus *A. fumigatus* relative to cells minus *A. fumigatus*.

### Gene Ontology and interaction network analyses

In order to better evaluate the complex patterns of up- and down-regulated genes and to compare the immune response after addition of moDC versus mDC, gene ontology and interaction network analyses were performed based on the lists of differentially expressed genes. Gene ontology (GO, over-representation of genes responsible for particular biological functions) analysis of the addition of DC and addition of *A. fumigatus* to the A549 cell layer led to an increase in the expression genes involved in chemotaxis, immune response and the inflammatory response (Table S1 and S2). By contrast the addition of mDC only increased the expression of genes involved in cell-to-cell signaling (Table S1), whereas the addition of moDC led to a response similar to that induced by *A. fumigatus* with genes involves in cell-to-cell signaling, chemotaxis, immune and inflammatory response (Table S2).

**Figure 3:**
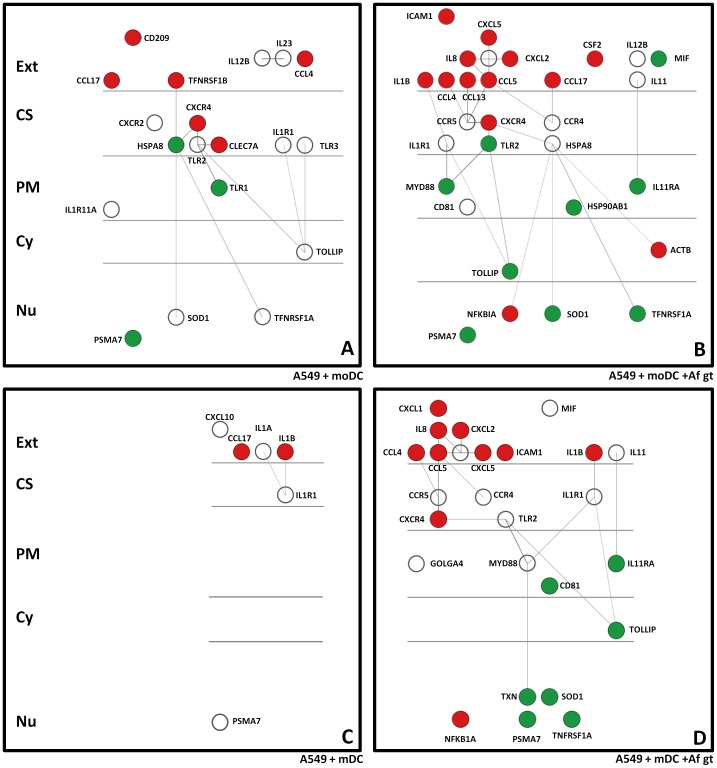
Gene network analyses of the expression data. Interaction networks identified when: (A) moDCs were added to the A549 cell layer. (B) moDCs and *A. fumigatus* germ tubes were added to the A549 layer. (C) mDCs were added to the A549 layer. (D) mDCs and *A. fumigatus* germ tubes were added to the A549 layer. The red circles indicate genes with an expression value >1.0; green circles had expression values <−1.0; white circles had expression less than 1.0 and greater than −1.0. The Network analyses were conducted through the Innate DB website and were visualized in Cytoscape. The predicted physical locations of expressed genes are also indicated: extracellular (Ext), cell surface (CS), plasma membrane (PM), cytoplasm (Cy) and nucleus (Nu).

The interaction network analysis (Figure 3) reflected the GO analysis in that the addition moDC in the absence of *A. fumigatus* led to a greater number of genes interacting in the absence of *A. fumigatus* compared to the addition of mDC. The data for both groups of DC in the presence of *A. fumigatus* was very similar. Both showed the up-regulation of pro-inflammatory cytokines and up-regulation of CXCR4. Overall there was an increase in the expression of genes related to secreted proteins and a reduction in intracellular signaling molecules such as Myd88. There was very limited activity in the A549 cell layer in the presence or absence of *A. fumigatus*.

## Discussion

The alveoli of the lung are usually the initial site for the establishment of infection by *A. fumigatus* [8]. They provide a nutrient rich environment that promotes the germination of conidia. Furthermore, the dense network of capillaries that carries blood to and from the alveoli provides a route for innate immune cells, including DC, into the lungs and for dissemination of the pathogen from the lungs [6]. *In vitro* models using human cell lines and primary human cells help to better understand the complex processes during these early stages of infection, including invasion of fungi into tissue and their local interaction with the host immune system.

Thus, we utilised a bilayer model [12] of the human alveolus to determine the changes in gene expression induced, in the structural cell layers of the alveolus and in DC subsets, within this system by *A. fumigatus*. We demonstrated that gene expression profiling experiments are possible with RNA extracted from this system, and that the bilayer model provides a useful tool to mimic the complex inflammatory situation when more than one cell type is involved. Furthermore, it allowed for the comparison of different immune cell subpopulations.

Both conidia and germ tubes induced the differential regulation of a limited number of genes in both the endothelial (HPAEC) or epithelial (A549) cell layers of the bilayer model (Tables 1 and 2). Only two genes were up-regulated (*GSK3A* and *SOD1*) in the A549 cell layer interacting with conidia and germ tubes. *GSK3A* plays a role in the immune response to fungi [24] but these data do not reflect gene expression analyses from A549 monolayers exposed to *A. fumigatus* [25]. It has been shown by RT- qPCR analysis that *IL8*, *TNF* and *CSF2* were differentially regulated by A549 cells exposed to *A. fumigatus* [25], however it is difficult to compare such studies since qPCR is more sensitive than microarrays, which can generally be seen in Fig. 2. However, qPCR is impractical for large numbers of genes.

Relatively modest fold changes in differentially expressed genes in epithelial cells have been reported for other microarray studies [26]. This may be because there was limited exposure of HPAEC cells to *A. fumigatus* since it takes approximately 16 h for the fungus to penetrate the alveolar-capillary boundary [6]. Thus, the experiments in this study focused on the interaction at the epithelial surface and were allowed to proceed for three to six hours. The limited damage caused by *A. fumigatus* when traversing cellular boundaries may also limit the extent of transcriptional responses in structural cell layers [6].

In contrast the addition of either mDC or moDC to the alveolar bilayer model led to a marked increase in the number of differentially regulated immune genes (Tables 5 and 6). The majority of studies examining the interaction between *A. fumigatus* and DC have used moDC [15], [16], [27] because they can be generated *in vitro* in relatively large quantities. This is one of the first studies to examine the interaction between *A. fumigatus* and human mDC. The moDC were more immunologically active than mDC in the absence of *A. fumigatus* which might be because moDC are differentiated from precursor monocytes through the addition of the cytokines IL-4 and GM-CSF. Primary immune cells such as mDC do not undergo this *ex vivo* differentiation and will be relatively un-stimulated by exogenous cytokines at the initiation of the experiment (Table 5). In addition, it has been postulated that moDC in the body may represent an auxillary inflammatory pathway whereas mDC are the specialized surveillance subset of DC [28].

Comparison of the expression profile from interactions of DC with *A. fumigatus* in planktonic culture (Tables 3 and 4) compared to interactions on the alveolar epithelium (Tables 5 and 6) revealed differences in the expression of *PTX3* and *CCL20* which are important for immune response against *A. fumigatus*. Pentraxin 3 is a soluble pathogen recognition receptor that is important for the phagocytosis and killing of *A. fumigatus* by immune cells since it acts as an opsonin [29]. It has recently been shown that mutations in *PTX3* may contribute to the risk of IA [30]. The difference in *CCL20* gene expression was only observed in mDC, not expressed in the presence of *A. fumigatus* in planktonic culture (Table 4); *CCL20* has been shown to enhance recruitment of inflammatory DCs to the lungs of neutropenic mice [21]. These results correspond to data which indicated that innate immune cells such as alveolar macrophages and neutrophils react to environmental dimensionality [31]. Specifically, phagocytosis of *A. fumigatus* was inhibited in 3-D growth models constructed from collagen. It may be expected that DCs on a model of the alveolar surface would react perform better than in planktonic culture which further emphasizes the importance of creating more accurate *in vitro* models to study host-pathogen interactions.

The gene for intercellular adhesion molecule 1 (*ICAM-1*) was only expressed when mDC and moDC interacted with the fungus on the alveolar model (Table 6), which suggested that the format of the interaction study may have an important effect on the behaviour of cells interacting with pathogenic fungi. It has been demonstrated that *A. fumigatus* can modulate vascular adhesion molecule (VCAM-1) in endothelial cells grown *in vitro* which has been suggested as a mechanism of enhancing host defence against angioinvasion through leukocute recruitment [32].

There was a significant increase in the number of immune genes up-regulated in mDC and moDC in response to *A. fumigatus* germ tubes (Table 6). This was in accordance with previous studies from our group using moDC in co-cultures with *A. fumigatus* germlings and subsequent Affymetrix array analysis [16] but also in experiments with the immune gene array used in this study [15]. In agreement with these studies *A. fumigatus* induced a pro-inflammatory response as indicated by the up-regulation of *IL1B*, *IL8* and *CCL20* (Table 6) and the up-regulation of pentraxin-3 (*PTX3*) gene expression, which was a characteristic response to fungal infection [33]. It is of interest that there was greater expression of *IL1B* in mDC (14× expression in mDC compared to 3× in moDC). This cytokine possesses a strong pro-inflammatory capacity with high potential for chemotaxis of monocytes and macrophages. In addition, IL1B, in synergy with IL23 is able to induce a Th17 response; both originate from an inactive IL1B precursor, pro-IL1B [34]. Polymorphisms in *IL1B* are associated with an increased risk of invasive pulmonary aspergillosis [35] further highlighting the importance of this cytokine in the anti-*Aspergillus* response.

Along with *IL1B*, both, *CXCL1* and *CCL23* showed greater expression in mDC than moDC (Table 6). These data indicate that mDC may possess a greater capacity for the expression of neutrophil chemoattractant cytokines than moDC. Furthermore, three additional chemokine genes (*CXCL2, CXCL5* and *CCL20*), which were markedly up-regulated in DC upon stimulation with *A. fumigatus* exhibit essential roles in neutrophil recruitment. CXCL5 has chemotactic and activating functions on neutrophils and is produced following stimulation of cells with the inflammatory cytokine IL-1B [36]; this cytokine was strongly up-regulated in our settings as well. CCL20 attracts neutrophils and dendritic cells, especially towards epithelial cells surrounding lymphoid tissues [37]. In addition to its neutrophil attraction, CXCL2 is able to trigger the *in vitro* adhesion of neutrophils to endothelial cells. Thereby, CXCL2 acts synergistically with ICAM-1 (Table 6), a strong mediator of leukocyte binding to endothelial cells, which is usually followed by their transmigration into tissues [38]. Taking the data from our alveolar model together, they underline the important role of DCs for the recruitment and adhesion of neutrophils to alveolar tissue.

In conclusion, we were able to demonstrate that this bilayer model, which reflects the human alveolus, allowed gene expression profiling of A549 cell layers interacting with human DC subpopulations and *A. fumigatus*. The system offers a feasible and robust approach for analyzing the interaction of human airway tissue with other cells or pathogens that complements animal models of infection. Thereby, interaction studies can be extended and in consequence this bilayer model can function as a basis for alternative approaches, including the analysis of other immune cell populations, further probe sets for alternative expression profiling (including miRNA profiling) and different respiratory pathogens. Improved understanding of the pathophysiology of *Aspergillus* infection, including local processes in human alveoli will help to understand the complexity of this infection and support the development of alternative therapeutic approaches.

## Materials and Methods

### Ethics Statement

This study, using whole blood specimens obtained from human healthy volunteer donors, was approved by the Ethical Committee of the University Hospital of Wuerzburg. Informed consent was written and provided by all study participants. Data analysis was conducted anonymously.

### Isolation of mDC and generation of moDC

Peripheral blood monocytes (PBMCs) were purified from donor buffy coat using MACS CD14 positive selection (Miltenyi Biotec) and induced to become immature dendritic cells (moDC) by incubation with IL-4 and GM-CSF [16]; moDC were generated from three different donors to 97% purity.

Myeloid DC (mDC) were purified from donor buffy coat using the CD1c (BDCA-1)^+^ dendritic cell isolation kit (Miltenyi Biotec) as described in the manufacturer's instructions. mDC were isolated from three different donors to 95% purity.

### Fungal strains and growth conditions

*Aspergillus fumigatus* strain Af293 was used in all experiments and cultures were maintained on malt extract agar. Suspensions of conidia were prepared as previously described and adjusted to a concentration of 2×10^5^ ml^−1^ in RPMI medium [15]. Germ tubes were generated by incubating conidia (2×10^5^ ml^−1^) overnight at room temperature in RPMI medium followed by incubation at 37°C for 6 h.

### Alveolar bilayer model

A model of the alveolar epithelium/endothelium was constructed on transwell membranes as described by Hope *et al.* [12]. Briefly, Human pulmonary artery endothelial cells (HPAEC) and Human alveolar epithelial cells (A549) were obtained from Lonza. The bilayer was constructed by incubating 1×10^5^ HPAECs on the under surface of a 6.5 mm diameter Transwell Clear membrane with 3-µM pores (Corning) for 2 h. The membrane was then placed right side up into the well of a 24-well tissue culture plate (Greiner) containing EGM-2 medium (Lonza), the upper chamber was filled with 100 ul EGM-2 medium and the assembly was incubated for 24 h at 37°C and 5% CO_2_. The contents of the upper chamber were removed and replaced by 5×10^4^ A549 cells in 100 µl EBM-2 plus 10% FBS, the assembly was then incubated at 37°C and 5% CO_2_. The respective media were changed daily and the experiment performed on the fifth day following addition of the A549 cells.

### Interaction experiments

Fungal elements, conidia or germ tubes (5×10^4^ per membrane), were added to the upper chamber of the transwell assembly to allow direct interaction of the fungus with the alveolar epithelium (A549 cell layer). Dendritic cells, either mDC or moDC, were added to the upper chamber at a concentration of 2×10^5^ per membrane. In interactions between the fungus and dendritic cells on the epithelium surface the MOI was one. Interactions were incubated for 3 h and 6 h at 37°C and 5% CO_2_.

### RNA extraction

After incubation each cell layer on the transwell membrane was removed using a cell scraper and pipette, the recovered cells were immediately transferred to eppendorf tubes containing buffer RLT (Qiagen). The A549 cell layer was transferred with the 200 µl of medium from the upper chamber to capture dendritic cells. RNA was extracted using the RNeasy mini kit (Qiagen) as per the manufacturer's protocol for RNA extraction from animal cells. After extraction the RNA was quantified and the RIN (RNA Integrity) value established using a 2100 Bioanalyzer (Agilent).

### Immune gene microarray

RNA from host cell layers was labelled and hybridised to a custom microarray of 117 oligos (Operon) targeting genes that are generally relevant for regulation of immune defence mechanisms (Genpak) [15]. This array has been listed on GEO under accession number GPL10270. This array was designed based on an Affymetrix array analysis of DC interacting with *A. fumigatus* [16]. This indicated that the selected immune genes showed the greatest differential regulation and had the most relevance to the interaction. This array reduced the complexity of analysis and allowed for potentially greater resolution of DC responses in the background of a second human cell type.

The experimental treatments tested were: (1) A549 cells plus conidia or germ tubes compared to uninfected A549 cells. (2) HPAEC cells in the bilayer model plus or minus A. fumigatus infection. (3) mDC or moDC plus conidia or germ tubes compared to uninfected cells. (4) A549 cells plus mDC or moDC compared to A549 cells with no added immune cells. (5) A549 cells plus mDC or moDC with germ tubes compared to uninfected A549 cells plus mDC or moDC.

Arrays were hybridised and analysed as previously described [15]. Briefly, 400 ng of total RNA was amplified using the “MessageAmp II aRNA Amplification Kit” from Ambion. The amplified aRNA from samples (stimulated and un-stimulated) were Cy3-labelled and a pool of all samples was Cy5-labelled by *in vitro* transcription from 500 ng of aRNA, respectively (LabelStar Array Kit, Qiagen). The gene chips were washed and probe arrays were scanned by using a GeneChip Scanner 3000 (Affymetrix).

### RT-qPCR validation of expression

For microarray validation by RT-qPCR, aliquots of the above mentioned RNA samples (500 ng) were reverse transcribed with the QuantiTect RT kit (Qiagen), containing a blend of oligo dT and random primers. RT-qPCR assays were performed using a StepOnePlus instrument (Applied Biosystems) using the TaqMan Universal PCR mix (Applied Biosystems) and commercially available primers and probes (Gene Expression assays) for *IL-1B, IL-8* and *CCL20*, with *ALAS1* as the endogenous control gene. All RT-PCR assays were run with an initial denaturation step (10 min at 95°C), followed by 40 cycles of repeated denaturation (15 s at 95°C) and primer annealing and extension (60 s at 60°C).

### Data Analysis

Data was analysed using software packages from the Bioconductor project (www.bioconductor.org; [39]) were run under R (www.r-project.org). After normalization (quantile-quantile) [40], differential expression of genes was calculated using the moderated t-statistic approach as implemented in the R-package Limma [41], which has been specifically developed for the analysis of small sample size experiments. By exploiting information across genes it delivers more stable results than a conventional t-test. The P-values of all results were corrected for multiple testing by application of the false discovery rate [42]. Correspondence analysis was performed using the routines implemented in the R-Package ‘vegan’[43].

### Gene ontology and network analysis

To facilitate interpretation the datasets were analysed by gene ontology and network analysis. The gene expression data for the interactions on the A549 cell layer induced by the addition of DCs and *A. fumigatus* germ tubes were uploaded to InnateDB (http://www.innatedb.com/). This is a comprehensive database and analysis platform that enables testing of differentially expressed genes in known immune response pathways. Gene Ontology and Network analyses were carried out using the default parameters (hyper-geometric test and Benjamini Hochberg correction for false discovery rate) [44], [45]. Networks of interacting genes were visualized in Cytoscape [46].

### Microarray accession numbers

The microarray data for the gene expression analyses in this study were submitted to the Gene Expression Omnibus (GEO, NCBI) under the accession number GSE28806.

## Supporting Information

Table S1 **Gene Ontology Analysis of A549 cells and mDC in the presence or absence of** ***A. fumigatus*** **germ tubes.**
(DOCX)

Table S2 **Gene Ontology Analysis of A549 cells and moDC in the presence or absence of** ***A. fumigatus*** **germ tubes.**
(DOCX)

## References

[1] Gugnani HC (2003) Ecology and taxonomy of pathogenic aspergilli. Front Biosci 8: s346–357. 12700046 10.2741/1002

[2] Latge JP (1999) Aspergillus fumigatus and aspergillosis. Clin Microbiol Rev 12: 310–350. 10194462 10.1128/cmr.12.2.310 PMC88920

[3] Hope WW, Walsh TJ, Denning DW (2005) The invasive and saprophytic syndromes due to Aspergillus spp. Med Mycol 43 Suppl 1S207–238. 16110814 10.1080/13693780400025179

[4] Neofytos D, Horn D, Anaissie E, Steinbach W, Olyaei A, et al (2009) Epidemiology and outcome of invasive fungal infection in adult hematopoietic stem cell transplant recipients: analysis of Multicenter Prospective Antifungal Therapy (PATH) Alliance registry. Clin Infect Dis 48: 265–273. 19115967 10.1086/595846

[5] Aimanianda V, Bayry J, Bozza S, Kniemeyer O, Perruccio K, et al (2009) Surface hydrophobin prevents immune recognition of airborne fungal spores. Nature 460: 1117–1121. 19713928 10.1038/nature08264

[6] Hope WW (2009) Invasion of the alveolar-capillary barrier by Aspergillus spp.: therapeutic and diagnostic implications for immunocompromised patients with invasive pulmonary aspergillosis. Med Mycol 47 Suppl 1S291–298. 19306226 10.1080/13693780802510232

[7] Park SJ, Mehrad B (2009) Innate immunity to Aspergillus species. Clinical microbiology reviews 22: 535–551. 19822887 10.1128/CMR.00014-09 PMC2772361

[8] Hope WW, Kruhlak MJ, Lyman CA, Petraitiene R, Petraitis V, et al (2007) Pathogenesis of Aspergillus fumigatus and the kinetics of galactomannan in an in vitro model of early invasive pulmonary aspergillosis: implications for antifungal therapy. The Journal of infectious diseases 195: 455–466. 17205486 10.1086/510535

[9] Schaller M, Mailhammer R, Korting HC (2002) Cytokine expression induced by Candida albicans in a model of cutaneous candidosis based on reconstituted human epidermis. Journal of medical microbiology 51: 672–676. 12171298 10.1099/0022-1317-51-8-672

[10] Schaller M, Korting HC, Borelli C, Hamm G, Hube B (2005) Candida albicans-secreted aspartic proteinases modify the epithelial cytokine response in an in vitro model of vaginal candidiasis. Infect Immun 73: 2758–2765. 15845479 10.1128/IAI.73.5.2758-2765.2005 PMC1087327

[11] Schaller M, Boeld U, Oberbauer S, Hamm G, Hube B, et al (2004) Polymorphonuclear leukocytes (PMNs) induce protective Th1-type cytokine epithelial responses in an in vitro model of oral candidosis. Microbiology 150: 2807–2813. 15347740 10.1099/mic.0.27169-0

[12] Hope WW, Kruhlak MJ, Lyman CA, Petraitiene R, Petraitis V, et al (2007) Pathogenesis of Aspergillus fumigatus and the kinetics of galactomannan in an in vitro model of early invasive pulmonary aspergillosis: implications for antifungal therapy. J Infect Dis 195: 455–466. 17205486 10.1086/510535

[13] Loeffler J, Haddad Z, Bonin M, Romeike N, Mezger M, et al (2009) Interaction analyses of human monocytes co-cultured with different forms of Aspergillus fumigatus. J Med Microbiol 58: 49–58. 19074652 10.1099/jmm.0.003293-0

[14] Cortez KJ, Lyman CA, Kottilil S, Kim HS, Roilides E, et al (2006) Functional genomics of innate host defense molecules in normal human monocytes in response to Aspergillus fumigatus. Infect Immun 74: 2353–2365. 16552065 10.1128/IAI.74.4.2353-2365.2006 PMC1418921

[15] Morton CO, Varga JJ, Hornbach A, Mezger M, Sennefelder H, et al (2011) The temporal dynamics of differential gene expression in Aspergillus fumigatus interacting with human immature dendritic cells in vitro. PLoS One 6: e16016. 21264256 10.1371/journal.pone.0016016 PMC3021540

[16] Mezger M, Kneitz S, Wozniok I, Kurzai O, Einsele H, et al (2008) Proinflammatory response of immature human dendritic cells is mediated by dectin-1 after exposure to Aspergillus fumigatus germ tubes. J Infect Dis 197: 924–931. 18279049 10.1086/528694

[17] Sugui JA, Kim HS, Zarember KA, Chang YC, Gallin JI, et al (2008) Genes differentially expressed in conidia and hyphae of Aspergillus fumigatus upon exposure to human neutrophils. PLoS One 3: e2655. 18648542 10.1371/journal.pone.0002655 PMC2481287

[18] McDonagh A, Fedorova ND, Crabtree J, Yu Y, Kim S, et al (2008) Sub-telomere directed gene expression during initiation of invasive aspergillosis. PLoS Pathog 4: e1000154. 18787699 10.1371/journal.ppat.1000154 PMC2526178

[19] Vermaelen K, Pauwels R (2005) Pulmonary dendritic cells. Am J Respir Crit Care Med 172: 530–551. 15879415 10.1164/rccm.200410-1384SO

[20] Bozza S, Gaziano R, Spreca A, Bacci A, Montagnoli C, et al (2002) Dendritic cells transport conidia and hyphae of Aspergillus fumigatus from the airways to the draining lymph nodes and initiate disparate Th responses to the fungus. J Immunol 168: 1362–1371. 11801677 10.4049/jimmunol.168.3.1362

[21] Park SJ, Burdick MD, Brix WK, Stoler MH, Askew DS, et al (2010) Neutropenia enhances lung dendritic cell recruitment in response to Aspergillus via a cytokine-to-chemokine amplification loop. J Immunol 185: 6190–6197. 20926800 10.4049/jimmunol.1002064 PMC3032263

[22] Tournier JN, Mohamadzadeh M (2010) Key roles of dendritic cells in lung infection and improving anthrax vaccines. Trends Mol Med 16: 303–312. 20554248 10.1016/j.molmed.2010.04.006

[23] Shetron-Rama LM, Herring-Palmer AC, Huffnagle GB, Hanna P (2010) Transport of Bacillus anthracis from the lungs to the draining lymph nodes is a rapid process facilitated by CD11c+ cells. Microb Pathog 49: 38–46. 20188814 10.1016/j.micpath.2010.02.004

[24] Spinnler K, Mezger M, Steffens M, Sennefelder H, Kurzai O, et al (2010) Role of glycogen synthase kinase 3 (GSK-3) in innate immune response of human immature dendritic cells to Aspergillus fumigatus. Medical mycology: official publication of the International Society for Human and Animal Mycology 48: 589–597. 10.3109/13693780903420625 20055739

[25] Bellanger AP, Millon L, Khoufache K, Rivollet D, Bieche I, et al (2009) Aspergillus fumigatus germ tube growth and not conidia ingestion induces expression of inflammatory mediator genes in the human lung epithelial cell line A549. J Med Microbiol 58: 174–179. 19141733 10.1099/jmm.0.005488-0

[26] Oosthuizen JL, Gomez P, Ruan J, Hackett TL, Moore MM, et al (2011) Dual organism transcriptomics of airway epithelial cells interacting with conidia of Aspergillus fumigatus. PLoS One 6: e20527. 21655222 10.1371/journal.pone.0020527 PMC3105077

[27] Gafa V, Lande R, Gagliardi MC, Severa M, Giacomini E, et al (2006) Human dendritic cells following Aspergillus fumigatus infection express the CCR7 receptor and a differential pattern of interleukin-12 (IL-12), IL-23, and IL-27 cytokines, which lead to a Th1 response. Infect Immun 74: 1480–1489. 16495518 10.1128/IAI.74.3.1480-1489.2006 PMC1418673

[28] Osugi Y, Vuckovic S, Hart DN (2002) Myeloid blood CD11c(+) dendritic cells and monocyte-derived dendritic cells differ in their ability to stimulate T lymphocytes. Blood 100: 2858–2866. 12351396 10.1182/blood.V100.8.2858

[29] Moalli F, Jaillon S, Inforzato A, Sironi M, Bottazzi B, et al (2011) Pathogen recognition by the long pentraxin PTX3. J Biomed Biotechnol 2011: 830421. 21716666 10.1155/2011/830421 PMC3118294

[30] Cunha C, Aversa F, Lacerda JF, Busca A, Kurzai O, et al (2014) Genetic PTX3 deficiency and aspergillosis in stem-cell transplantation. N Engl J Med 370: 421–432. 24476432 10.1056/NEJMoa1211161

[31] Behnsen J, Narang P, Hasenberg M, Gunzer F, Bilitewski U, et al (2007) Environmental dimensionality controls the interaction of phagocytes with the pathogenic fungi Aspergillus fumigatus and Candida albicans. PLoS Pathog 3: e13. 17274685 10.1371/journal.ppat.0030013 PMC1790725

[32] Chiang LY, Sheppard DC, Gravelat FN, Patterson TF, Filler SG (2008) Aspergillus fumigatus stimulates leukocyte adhesion molecules and cytokine production by endothelial cells in vitro and during invasive pulmonary disease. Infect Immun 76: 3429–3438. 18490455 10.1128/IAI.01510-07 PMC2493209

[33] Gaziano R, Bozza S, Bellocchio S, Perruccio K, Montagnoli C, et al (2004) Anti-Aspergillus fumigatus efficacy of pentraxin 3 alone and in combination with antifungals. Antimicrob Agents Chemother 48: 4414–4421. 15504871 10.1128/AAC.48.11.4414-4421.2004 PMC525434

[34] Romani L (2008) Cell mediated immunity to fungi: a reassessment. Med Mycol 46: 515–529. 19180748 10.1080/13693780801971450

[35] Ok M, Einsele H, Loeffler J (2011) Genetic susceptibility to Aspergillus fumigatus infections. Int J Med Microbiol 301: 445–452. 21550849 10.1016/j.ijmm.2011.04.013

[36] Hieshima K, Imai T, Opdenakker G, Van Damme J, Kusuda J, et al (1997) Molecular cloning of a novel human CC chemokine liver and activation-regulated chemokine (LARC) expressed in liver. Chemotactic activity for lymphocytes and gene localization on chromosome 2. J Biol Chem 272: 5846–5853. 9038201 10.1074/jbc.272.9.5846

[37] Chang MS, McNinch J, Basu R, Simonet S (1994) Cloning and characterization of the human neutrophil-activating peptide (ENA-78) gene. J Biol Chem 269: 25277–25282. 7929219

[38] Yang L, Froio RM, Sciuto TE, Dvorak AM, Alon R, et al (2005) ICAM-1 regulates neutrophil adhesion and transcellular migration of TNF-alpha-activated vascular endothelium under flow. Blood 106: 584–592. 15811956 10.1182/blood-2004-12-4942 PMC1635241

[39] Gentleman RC, Carey VJ, Bates DM, Bolstad B, Dettling M, et al (2004) Bioconductor: open software development for computational biology and bioinformatics. Genome Biol 5: R80. 15461798 10.1186/gb-2004-5-10-r80 PMC545600

[40] Smyth GK, Speed TP (2003) Normalization of cDNA microarray data. Methods 31: 265–273. 14597310 10.1016/s1046-2023(03)00155-5

[41] Smyth GK (2004) Linear models and empirical bayes methods for assessing differential expression in microarray experiments. Statistical applications in genetics and molecular biology 3: Article3. 16646809 10.2202/1544-6115.1027

[42] Benjamini Y, Hochberg Y (1995) Controlling the false discovery rate: a practical and powerful approach to multiple testing. J Roy Stat Soc B Met 57: 289–300.

[43] Oksanen J, Blanchet FG, Kindt R, Legendre P, O'Hara RB, et al.. (2011) vegan: Community Ecology Package.

[44] Ballarin A, Bazzan E, Zenteno RH, Turato G, Baraldo S, et al (2012) Mast cell infiltration discriminates between histopathological phenotypes of chronic obstructive pulmonary disease. American journal of respiratory and critical care medicine 186: 233–239. 22679009 10.1164/rccm.201112-2142OC

[45] Lynn DJ, Winsor GL, Chan C, Richard N, Laird MR, et al (2008) InnateDB: facilitating systems-level analyses of the mammalian innate immune response. Mol Syst Biol 4: 218. 18766178 10.1038/msb.2008.55 PMC2564732

[46] Hanley PJ, Kronlage M, Kirschning C, del Rey A, Di Virgilio F, et al (2012) Transient P2X7 receptor activation triggers macrophage death independent of Toll-like receptors 2 and 4, caspase-1, and pannexin-1 proteins. The Journal of biological chemistry 287: 10650–10663. 22235111 10.1074/jbc.M111.332676 PMC3323034

