# Supplementary material for: Gene Expression Profiles of Human Dendritic Cells Interacting with Aspergillus fumigatus in a Bilayer Model of the Alveolar Epithelium/Endothelium Interface
Source: PLoS One. 2014 May 28;9(5):e98279. doi: 10.1371/journal.pone.0098279 (PMC4037227; doi:10.1371/journal.pone.0098279)
Supplement: Table S1 — Gene Ontology Analysis of A549 cells and mDC in the presence or absence of A. fumigatus germ tubes. (DOCX) [file pone.0098279.s001.docx]

**Table S1**. Gene Ontology Analysis of A549 cells and mDC in the presence or absence of *A. fumigatus* germ tubes. **GO Terms with mainly Up-regulated Genes**

| **Minus *A. fumigatus*** | | | | | **Plus *A. fumigatus*** | | | |
| --- | --- | --- | --- | --- | --- | --- | --- | --- |
| **Cell-cell signalling (p: 0.08)** | | | | | **Chemotaxis (p: 0.006)** | | | |
| **Gene ID** | **Gene Annotation** | | | **Fold Change** | **Gene ID** | **Gene Annotation** | **Fold Change** | |
| CCL17  CXCL5  IL1B  CXCL10 | Chemokine (C-C-Motif) Ligand 17  Chemokine (C-X-C-Motif) Ligand 5  Interleukin-1 beta  Chemokine (C-X-C-Motif) Ligand 10 | | | 3.8  1.2  1.3  -0.6 | CCL20 CCL23 CCL4 CCL5 CCR4 CCR5 CXCL1 CXCL2 CXCL5 | Chemokine (C-C-Motif) Ligand 20  Chemokine (C-C-Motif) Ligand 23  Chemokine (C-C-Motif) Ligand 4  Chemokine (C-C-Motif) Ligand 5  Chemokine (C-C-Motif) Receptor 4  Chemokine (C-C-Motif) Receptor 5  Chemokine (C-X-C-Motif) Ligand 1  Chemokine (C-X-C-Motif) Ligand 2  Chemokine (C-X-C-Motif) Ligand 5 | 4.2  1.1  3.3  1.1  -0.9  -0.6  3  4  3.3 | |
|  | | | | | **Immune response (p: 0.006)** | | | |
|  |  | | |  | CCL19 CCL20 CCL21 CCL23 CCL4  CCL5  CCR4  CCR5  CXCL1 CXCL2 CXCL3 CXCL5  IL1B  IL1R1  IL8  TLR2 | Chemokine (C-C-Motif) Ligand 19  Chemokine (C-C-Motif) Ligand 20  Chemokine (C-C-Motif) Ligand 21  Chemokine (C-C-Motif) Ligand 23  Chemokine (C-C-Motif) Ligand 4  Chemokine (C-C-Motif) Ligand 5  Chemokine (C-C-Motif) Receptor 4  Chemokine (C-C-Motif) Receptor 5  Chemokine (C-X-C-Motif) Ligand 1  Chemokine (C-X-C-Motif) Ligand 2  Chemokine (C-X-C-Motif) Ligand 3  Chemokine (C-X-C-Motif) Ligand 5  Interleukin-1 beta  Interleukin-1 Receptor 1  Interleukin-8  Toll-like Receptor 2 | | -0.9  4.2  -0.8  1.1  3.3  1.1  -0.9  -0.6  3  4  2.2  3.2  3.8  -0.5  1.8  -0.9 |
|  | | | | | **Negative regulation of cell proliferation**  **(p: 0.013)** | | | |
|  | |  |  | | CCL23 CXCL1  IL1B  IL8 | Chemokine (C-C-Motif) Ligand 23  Chemokine (C-X-C-Motif) Ligand 1  Interleukin-1 beta  Interleukin-8 | | 1.1  3  3.8  1.8 |
|  | | | | | **Inflammatory response (p: 0.016)** | | | |
|  | |  |  | | CCL19 CCL20 CCL21 CCL23 CCL4  CCL5  CCR4  CCR5 CXCL1 CXCL2 CXCL3 CXCR4  IL1B  IL8  MIF  MYD88  PTX3  TLR2 TNFRSF1A  TOLLIP | Chemokine (C-C-Motif) Ligand 19  Chemokine (C-C-Motif) Ligand 20  Chemokine (C-C-Motif) Ligand 21  Chemokine (C-C-Motif) Ligand 23  Chemokine (C-C-Motif) Ligand 4  Chemokine (C-C-Motif) Ligand 5  Chemokine (C-C-Motif) Receptor 4  Chemokine (C-C-Motif) Receptor 5  Chemokine (C-X-C-Motif) Ligand 1  Chemokine (C-X-C-Motif) Ligand 2  Chemokine (C-X-C-Motif) Ligand 3  Chemokine (C-X-C-Motif) Receptor 4  Interleukin-1 beta  Interleukin-8  Macrophage migration inhibitory factor  Myeloid differentiation primary response gene (88)  Pentraxin 3  Toll-like Receptor 2  Tumour necrosis factor-Receptor Superfamily 1A  Toll interacting protein | | -0.9  4.2  -0.8  1.1  3.3  1.1  -0.9  -0.6  3  4  2.2  1.1  3.8  1.8  -0.9  -0.8  1.2  -0.9  -2.8  -1.4 |
|  | | | | | **Neutrophil chemotaxis (p: 0.043)** | | | |
|  | |  |  | | CXCL3  IL1B  IL8 | Chemokine (C-X-C-Motif) Ligand 3  Interleukin-1 beta  Interleukin-8 | | 2.2  3.8  1.8 |
