# Supplementary material for: Gene Expression Profiles of Human Dendritic Cells Interacting with Aspergillus fumigatus in a Bilayer Model of the Alveolar Epithelium/Endothelium Interface
Source: PLoS One. 2014 May 28;9(5):e98279. doi: 10.1371/journal.pone.0098279 (PMC4037227; doi:10.1371/journal.pone.0098279)
Supplement: Table S2 — Gene Ontology Analysis of A549 cells and moDC in the presence or absence of A. fumigatus germ tubes. (DOCX) [file pone.0098279.s002.docx]

**Table S2**. Gene Ontology Analysis of A549 cells and moDC in the presence or absence of *A. fumigatus* germ tubes. **GO Terms with mainly Up-regulated Genes**

| **Minus *A. fumigatus*** | | | | | | | | **Plus *A. fumigatus*** | | | |
| --- | --- | --- | --- | --- | --- | --- | --- | --- | --- | --- | --- |
| **Cell-cell signaling (p: 0.08)** | | | | | | | | **Chemotaxis (p: 0.008)** | | | |
| **Gene ID** | | | **Gene Annotation** | | | **Fold Change** | | **Gene ID** | **Gene Annotation** | **Fold Change** | |
| CCL13 CCL17 CCL18 CCL23 CCL4 TOLLIP | | | Chemokine (C-C-Motif) Ligand 13  Chemokine (C-C-Motif) Ligand 17  Chemokine (C-C-Motif) Ligand 18  Chemokine (C-C-Motif) Ligand 23  Chemokine (C-C-Motif) Ligand 4  Toll interacting protein | | | 4.9  3  5.3  1.7  1.1  -0.5 | | CCL13CCL17CCL18CCL20CCL4  CCL5  CCR4  CCR5  CXCL2CXCL5 | Chemokine (C-C-Motif) Ligand 13  Chemokine (C-C-Motif) Ligand 17  Chemokine (C-C-Motif) Ligand 18  Chemokine (C-C-Motif) Ligand 20  Chemokine (C-C-Motif) Ligand 4  Chemokine (C-C-Motif) Ligand 5  Chemokine (C-C-Motif) Receptor 4  Chemokine (C-C-Motif) Receptor 5  Chemokine (C-X-C-Motif) Ligand 2  Chemokine (C-X-C-Motif) Ligand 5 | 2.6  1  2.4  3  5.2  1  -0.6  -0.5  3.3  2.1 | |
| **Chemotaxis (p: 0.008)** | | | | | | | | **Immune response (p: 0.006)** | | | |
| CCL13 CCL17 CCL18 CCL23 CCL4 | Chemokine (C-C-Motif) Ligand 13  Chemokine (C-C-Motif) Ligand 17  Chemokine (C-C-Motif) Ligand 18  Chemokine (C-C-Motif) Ligand 23  Chemokine (C-C-Motif) Ligand 4 | | | | | 4.9  3  5.3  1.7  1.1 | | CCL13CCL17CCL18CCL19CCL20CCL4  CCL5  CCR4  CCR5  CSF2  CXCL2CXCL3CXCL5  IL10RBIL1B  IL1R1  IL8  TLR2 | Chemokine (C-C-Motif) Ligand 13  Chemokine (C-C-Motif) Ligand 17  Chemokine (C-C-Motif) Ligand 18  Chemokine (C-C-Motif) Ligand 19  Chemokine (C-C-Motif) Ligand 20  Chemokine (C-C-Motif) Ligand 4  Chemokine (C-C-Motif) Ligand 5  Chemokine (C-C-Motif) Receptor 4  Chemokine (C-C-Motif) Receptor 5  Colony stimulating factor 2 (granulocyte-macrophage)  Chemokine (C-X-C-Motif) Ligand 2  Chemokine (C-X-C-Motif) Ligand 3  Chemokine (C-X-C-Motif) Ligand 5  Interleukin-10 Receptor B  Interleukin-1 beta  Interleukin-1 Receptor 1  Interleukin-8  Toll-like Receptor 2 | | 2.6  1  2.4  -0.7  3  5.2  1  -0.6  -0.5  1.3  3.3  1  2.1  -0.6  1.3  -0.6  2.2  -1.5 |
| **Immune response (p: 0.034)** | | | | | | | | **Cell-cell signalling (p: 0.02)** | | | |
| CCL13 CCL17 CCL18 CCL23  CCL4  IL1R1  IL23  TLR1  TLR2 TNFRSF1B | | Chemokine (C-C-Motif) Ligand 13  Chemokine (C-C-Motif) Ligand 17  Chemokine (C-C-Motif) Ligand 18  Chemokine (C-C-Motif) Ligand 23  Chemokine (C-C-Motif) Ligand 4  Interleukin-1 Receptor 1  Interleukin-23  Toll-like Receptor 1  Toll-like Receptor 2  Tumour necrosis factor-Receptor Superfamily 1B | | | | 4.9  3  5.3  1.7  1.1  -0.7  -0.6  -1.1  -0.7  1.1 | | CCL13CCL17CCL18CCL20CCL21CCL4  CCL5  CCR5  CXCL5  IL11  IL1B  TOLLIP | Chemokine (C-C-Motif) Ligand 13  Chemokine (C-C-Motif) Ligand 17  Chemokine (C-C-Motif) Ligand 18  Chemokine (C-C-Motif) Ligand 20  Chemokine (C-C-Motif) Ligand 21  Chemokine (C-C-Motif) Ligand 4  Chemokine (C-C-Motif) Ligand 5  Chemokine (C-C-Motif) Receptor 5  Chemokine (C-X-C-Motif) Ligand 5  Interleukin-11  Interleukin-1 beta  Toll interacting protein | | 2.6  1  2.4  3  -0.7  5.2  1  -0.5  2.1  -0.9  1.3  -1.3 |
| **Inflammatory response (p: 0.05)** | | | | | | | | **Inflammatory response (p: 0.039)** | | | |
| CCL13 CCL17 CCL18 CCL23  CCL4 Dectin-1 CXCR4 IL23  TLR1  TLR2  TLR3  TLR5  TLR7  TNFRSF1A  TNFRSF1B  TOLLIP | | Chemokine (C-C-Motif) Ligand 13  Chemokine (C-C-Motif) Ligand 17  Chemokine (C-C-Motif) Ligand 18  Chemokine (C-C-Motif) Ligand 23  Chemokine (C-C-Motif) Ligand 4  Dectin-1  Chemokine (C-X-C-Motif) Receptor 4  Interleukin-23  Toll-like Receptor 1  Toll-like Receptor 2  Toll-like Receptor 3  Toll-like Receptor 5  Toll-like Receptor 7  Tumour necrosis factor-Receptor Superfamily 1A  Tumour necrosis factor-Receptor Superfamily 1B  Toll interacting protein | | | | | 4.9  3  5.3  1.7  1.1  1.6  1.5  -0.6  -1.1  -0.7  -0.9  -1.1  -1.2  -0.8  1.1  -0.5 | CCL13CCL17CCL18 CCL19 CCL20 CCL21 CCL4 CCL5 CCR4 CCR5 CXCL2 CXCL3 CXCR4 IL10RB IL1B IL8 MIF  MYD88  PTX3 TLR2 TNFRSF1A  TOLLIP | Chemokine (C-C-Motif) Ligand 13  Chemokine (C-C-Motif) Ligand 17  Chemokine (C-C-Motif) Ligand 18  Chemokine (C-C-Motif) Ligand 19  Chemokine (C-C-Motif) Ligand 20  Chemokine (C-C-Motif) Ligand 21  Chemokine (C-C-Motif) Ligand 4  Chemokine (C-C-Motif) Ligand 5  Chemokine (C-C-Motif) Receptor 4  Chemokine (C-C-Motif) Receptor 5  Chemokine (C-X-C-Motif) Ligand 2 Chemokine (C-X-C-Motif) Ligand 3  Chemokine (C-X-C-Motif) Receptor 4  Interleukin-10 Receptor B  Interleukin-1 beta  Interleukin-8  Macrophage migration inhibitory factor  Myeloid differentiation primary response gene (88)  Pentraxin 3  Toll-like Receptor 2  Tumour necrosis factor-Receptor Superfamily 1A  Toll interacting protein | | 2.6  1  2.4  -0.7  3  -0.7  5.2  1  -0.6  -0.5  3.3  1  1.8  -0.6  1.3  2.2  -1.4  -1.3  1.3  -1.5  -2.6  -1.3 |
|  | | | | | | | | **Neutrophil chemotaxis (p: 0.05)** | | | |
|  | | | |  |  | | | CXCL3 IL1B  IL8 | Chemokine (C-X-C-Motif) Ligand 3  Interleukin-1 beta  Interleukin-8 | | 1  1.3  2.2 |
